# Utilizing Zirconium MOF‐functionalized Fiber Substrates Prepared by Molecular Layer Deposition for Toxic Gas Capture and Chemical Warfare Agent Degradation

**DOI:** 10.1002/gch2.202100001

**Published:** 2021-09-12

**Authors:** Agnieszka Gorzkowska‐Sobas, Kristian Blindheim Lausund, Martijn C. de Koning, Veljko Petrovic, Sachin M. Chavan, Martin W. Smith, Ola Nilsen

**Affiliations:** ^1^ Norwegian Defence Research Institute FFI Instituttveien 21 Kjeller 2007 Norway; ^2^ Centre for Materials Science and Nanotechnology Department of Chemistry University of Oslo Sem Sælands vei 26 Oslo 0371 Norway; ^3^ TNO Lange Kleiweg 137 2288 GJ, Rijswijk The Netherlands; ^4^ Department of Chemistry Bioscience and Environmental Engineering University of Stavanger Stavanger 4036 Norway; ^5^ CBR Division Defence Science & Technology Laboratory Porton Down Salisbury SP4 0JQ UK

**Keywords:** degradation, metal–organic frameworks, nerve agents, protective equipment, thin films, toxic gases

## Abstract

Metal–organic frameworks (MOFs) are a class of porous organic‐inorganic solids extensively explored for numerous applications owing to their catalytic activity and high surface area. In this work MOF thin films deposited in a one‐step, molecular layer deposition (MLD), an all‐gas‐phase process, on glass wool fibers are characterized by X‐ray diffraction, Fourier transform infrared spectroscopy, scanning electron microscopy, and their capabilities towards toxic industrial chemical (TIC) capture and chemical warfare agents (CWA) degradation are investigated. It is shown that despite low volume of the active material used, MOFs thin films are capable of removal of harmful gaseous chemicals from air stream and CWA from neutral aqueous environment. The results confirm that the MLD‐deposited MOF thin films, amorphous and crystalline, are suitable materials for use in air filtration, decontamination, and physical protection against CWA and TIC.

## Graphical abstract

Glass fibers coated with amorphous and crystalline amino‐functionalized Zr‐metal‐organic‐frameworks (Zr‐MOF) films show activity towards capturing NH_3_ and enabling the catalytic degradation of nerve agents. A novel, one‐step molecular layer deposition method is used for direct fiber coating. This indicates the potential of Zr‐MOF functionalized fibrous materials for improved decontamination methods and protective equipment against various toxic chemicals.

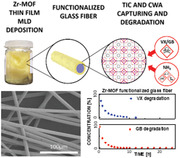

## 1 Introduction

Metal–organic frameworks (MOFs) are a class of porous organic‐inorganic solids, where metal‐based clusters are connected to each other via organic linkers to form a wide variety of networks, including complex 3D structures. Owing to their unique, organic‐inorganic nature and exceptionally high internal surface area, MOFs are promising candidates for many applications, especially in the fields of adsorption (gas separation, air purification) and catalysis. Recently, it has been demonstrated that MOFs not only are capable of efficiently capturing many Toxic Industrial Chemicals (TICs), but are also able to degrade the most toxic chemicals known to humankind, such as Chemical Warfare Agents (CWAs).^[^ ^1^, ^2^, ^3^, ^4^, ^5^, ^6^, ^7^, ^8^, ^9^, ^10^, ^11^, ^12^, ^13^, ^14^, ^15^, ^16^, ^17^, ^18^, ^19^, ^20^, ^21^, ^22^, ^23^, ^24^, ^25^, ^26^, ^27^, ^28^, ^29^, ^30^, ^31^, ^32^ ^]^ These phenomena are important for development of more efficient and versatile technological solutions in the field of individual protective equipment (IPE) against TICs and CWAs, such as respirator filters and protective clothing. Moreover, it could well contribute to decontaminating capabilities. Thus, use of MOFs might offer a significant benefit of combining air purification and decontamination functionalities. In this respect, zirconium‐MOFs (Zr‐MOFs) are particularly good candidates due to their exceptional stability and excellent performance in catalytic degradation of CWAs, especially towards nerve agents (e.g., Sarin (GB), VX, tabun (GA), soman (GD), and their simulants).^[^ ^2^, ^22^, ^23^, ^24^, ^33^ ^]^

However, most commercially available and laboratory synthesized MOFs are in the form of fine powders, which hinders their practical use in many applications. For instance, in the case of respiratory protection devices, a fine powder sorbent would lead to a significant increase in airflow resistance across the filter. Analogously, the use of MOFs as a protective layer in clothing requires some form of controlled immobilization of the MOFs on a carrier material. Therefore, a considerable effort has been put into immobilization of MOFs on different substrates, including fibers,^[^ ^20^, ^26^, ^34^, ^35^, ^36^ ^]^ woven and non‐woven fabrics,^[^ ^14^, ^16^, ^27^, ^30^, ^34^ ^]^ meshes,^[^ ^37^ ^]^ and even as self‐supporting structures.^[^ ^18^, ^38^ ^]^ Nonetheless, most techniques usually require a multi‐step deposition process, where the MOF is either synthesized prior to the deposition,^[^ ^14^, ^27^, ^29^, ^35^ ^]^ or grown in situ by wet chemical techniques, often on a specially prepared seeding layer.^[^ ^3^, ^26^, ^29^, ^30^, ^39^ ^]^ An alternative approach to immobilize MOFs on a surface is to synthesize MOFs directly on the substrate using gaseous precursors. Recently, Lausund et al.^[^ ^40^, ^41^, ^42^ ^]^ have shown that Zr‐MOFs can be successfully deposited on silicon substrates in an all‐gas‐phase, one‐step procedure, using a technique known as molecular layer deposition (MLD). Specifically, the MLD deposition process of amino‐functionalized Zr‐MOF was developed by Lausund et al.^[^ ^41^ ^]^ and utilized for flat Si substrates. MLD is a cyclic deposition technique that is closely related to atomic layer deposition (ALD), where a material is synthesized through sequential exposure of a substrate by two or more gaseous precursors. The reactions are self‐limiting and ensure even film on all available surfaces, such as fibers and textiles.^[^ ^43^, ^44^ ^]^

In this work, we take a step further and explore the properties of Zr‐MOF thin films for adsorption of TIC and degradation of CWA. For this purpose, amino‐functionalized Zr‐MOF has been deposited in a one‐step, all‐gas‐phase MLD process on a model fibrous substrate—a non‐treated glass wool, being an inert, inexpensive material already used in particle filter manufacturing. We demonstrate the activity of the fibers coated with amorphous and crystalline amino‐functionalized Zr‐MOF films by capturing ammonia (NH_3_), a highly volatile TIC, and enabling the catalytic degradation of two structurally different CWAs: sarin (GB) and VX that belong to different subclasses of nerve agents (G‐agents and V‐agents, respectively).^[^ ^45^ ^]^

## 2 Results and Discussion

As described in the experimental section, a glass wool piece was placed in the ALD reactor and coated with ≈270 nm of Zr‐MOF film. The thickness of the as‐deposited films was estimated by averaging the film thickness on the two Si substrates placed in the reactor (upstream = 399 nm, downstream = 146 nm, see the experimental section for details). The observed film growth on the substrate placed downstream from the glass wool indicates that the precursors have been able to access the entire volume of the reactor, coating the fibers within the glass wool piece. The thickness gradient can be ascribed to either a component of chemical vapor deposition‐type growth upstream in the reactor due to a restriction of the flow of precursors, a loss of film downstream in the reactor due to etching of the film by the HCl byproduct, or a combination of these two effects.

**Figure 1:**
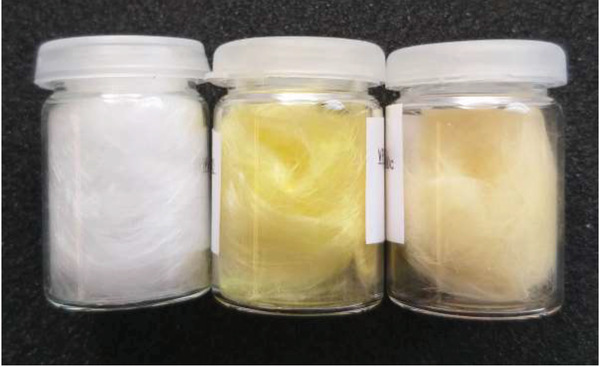
A photograph of the uncoated glass wool, as‐deposited samples, and crystallized samples (from left to right). The yellow color is characteristic of UiO‐66‐NH_2_.

The coated glass wool fibers gained a distinct yellow color characteristic of UiO‐66‐NH_2_ (**Figure** **1**). Upon handling, the fibers appeared to be more rigid after deposition, and could easily be separated from each other, indicating that the film covers each individual fiber evenly without binding them together. These features are also seen for the acetic acid treated samples, although a slight color change is observed, probably resulting from a change in light scattering by the material due to the development of the grain boundaries between UiO‐66‐NH_2_ crystallites.

**Figure 2:**
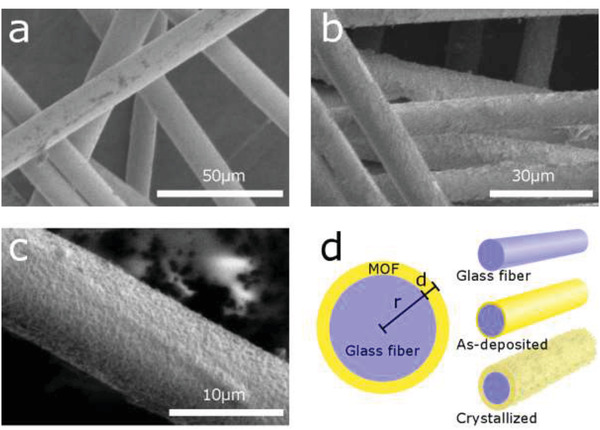
Scanning electron microscope images of a) as‐deposited MOF coated glass fibers, b) glass fibers coated with a crystallized MOF film, and c) a glass fiber coated with crystallized film at higher magnification. d) Schematic illustration of a neat glass fiber, an as‐deposited sample, a crystallized sample, and the cross section of the as‐deposited sample (not to scale).

The surface of the coated fibers was further examined using SEM (**Figure** **2**). The as‐deposited samples have a smooth, almost featureless surface (Figure 2a), while the surface of crystallized samples (Figure 2b,c) looks coarse, resembling orange peel, similarly to that observed for the UiO‐66‐NH_2_ films deposited on silicon substrates in previous work.^[^ ^41^ ^]^ Some areas of the film show partial delamination, leaving the exposed glass wool surface and loose flakes of the material around the fibers (Figure S1b, Supporting Information). This damage might be caused by mechanical handling of the samples, such as pressing against the carbon tape on the SEM holders, indicating a need for improved film adhesion to the substrate.

**Figure 3:**
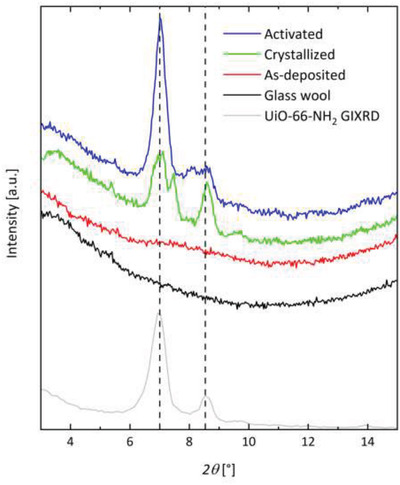
X‐ray diffractograms for the as‐deposited, crystallized, and activated samples compared to uncoated glass wool and a UiO‐66‐NH_2_ film on a silicon substrate as references.

The samples were further characterized by XRD measurements. The diffraction patterns for our samples are shown in **Figure** **3** (full diffractograms are shown in Figure S2, Supporting Information). For reference, diffractogram obtained by grazing incidence X‐ray diffraction of uncoated glass fibers and a crystalline UiO‐66‐NH_2_ film deposited by MLD on a Si wafer^[^ ^41^ ^]^ were included.

The as‐deposited sample shows no sharp reflections owing to the films amorphous nature. However, when compared to the uncoated glass fiber, a small “bump” at low 2θ values (≈6–10°) can be seen. Similar XRD patterns have previously been observed for amorphous MOF materials.^[^ ^46^, ^47^ ^]^ The reflections at ≈7° and 8.5° emerging after the acetic acid treatment correspond to the main intensity peaks for UiO‐66‐NH_2_, thus confirming that the treatment resulted in material crystallization. These reflections became even more pronounced for the scCO_2_ activated sample. It is likely that removal of the acetic acid from the pores leads to a further ordering of the structure, where the random reflections from acetic acid are eliminated.

The chemical composition and structure of the MOF films were also examined by FTIR measurements (Figure S3, Supporting Information). The presence of well‐defined peaks with the maximum in the carboxylate region indicates that the post‐deposition treatment with acetic acid promoted crystallization of the film. This is further confirmed by the magnitude of the separation between the carboxylate peaks indicating a bridging bidentate coordination between the carboxylate group and two Zr‐atoms, which is expected in UiO‐type MOFs.^[^ ^48^ ^]^

We also attempted to measure nitrogen adsorption isotherms of the activated samples (Figure S4, Supporting Information). The total nitrogen uptake of the sample was rather low, although significantly larger than that of the uncoated glass wool substrate. By assuming an even distribution of the 270 nm thick MOF layer along the approximately 7.5 µm thick fibers, a glass fiber density of 2.65 g^−1^ cm^3^ (equal to a pure SiO_2_‐glass^[^ ^49^ ^]^), and a MOF density of ≈2.3 g^−1^ cm^3^, as measured by X‐ray reflectivity,^[^ ^41^ ^]^ we were able to calculate a MOF loading of 11.5 wt%, which is a relatively low value. Furthermore, some of the MOF layer could be lost due to mechanical handling of the sample, as revealed by some of the SEM images (Figure S1, Supporting Information). This could account for the observed low adsorption per sample weight, since several mg of sample material typically are required for measuring an accurate N_2_ adsorption isotherm.

**Figure 4:**
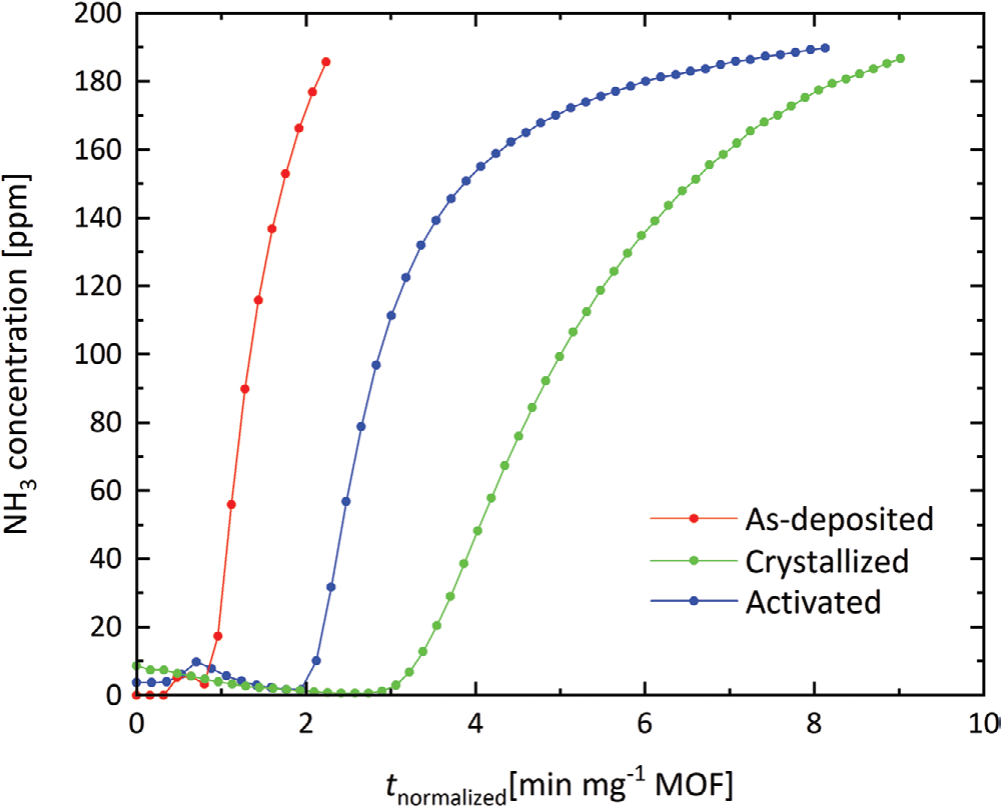
NH_3_ breakthrough curves for the as‐deposited, crystallized, and activated samples.

Having successfully coated the glass fibers with MOF, further testing focused on demonstrating their potential application in protective concepts. First, the interaction with NH_3_ was investigated. The adsorption curves of the NH_3_ in the MOF‐covered glass fibers measured under dynamic conditions are shown in **Figure** **4** (breakthrough curves). The curves were collected for the MOF‐coated fibers exposed to a challenge of 200 ppm NH_3_ in N_2_ as the carrier gas. The shortest breakthrough time was observed for the as‐deposited sample, reflecting its low adsorption capacity towards NH_3_, as expected for a low crystallinity material. Subsequent treatment with acetic acid clearly had a beneficial effect on the sample performance, as shown by the increased NH_3_ breakthrough time. In comparison, for the uncoated glass wool, the breakthrough is almost immediate (Figure S5, Supporting Information).

The adsorption capacity at the breakthrough time (A_bt_) values for the MOF coatings are given in **Table** **1**. As can be seen, all the samples are capable of NH_3_ adsorption, regardless of their crystalline structure, thus indicating their porosity. Interestingly, the non‐activated sample had an even higher adsorption capacity than the activated one. This could be ascribed to active participation of residual acetic acid present in the pores of the non‐activated material, presumably by acid‐base interactions. However, the exact cause of this effect is still to be investigated. The *A*_bt_ values are also lower than those for the benchmark UiO‐66‐NH_2_ in bulk form (powder) found in literature, where values over 3 mmol NH_3_ g^−1^ MOF were reported.^[^ ^6^, ^8^ ^]^ In our case, the incomplete conversion of the films into crystalline material, as well as loss of the film during handling are the important factors affecting the adsorption capacity values. Still, the results are valuable to assess the sample performance towards NH_3_ adsorption, even though the adsorption capacities obtained from a single breakthrough time value (corresponding to a single point on the isotherm) are expected to be lower than those obtained from breakthrough curve integration.

**Table 1:** Adsorption capacity of the MOF films towards NH_3_

Sample	*A*_bt_ (mmol NH_3_ g^−1^ MOF coating)
As‐deposited	0.11
Crystallized	0.53
Activated	0.32

**Figure 5:**
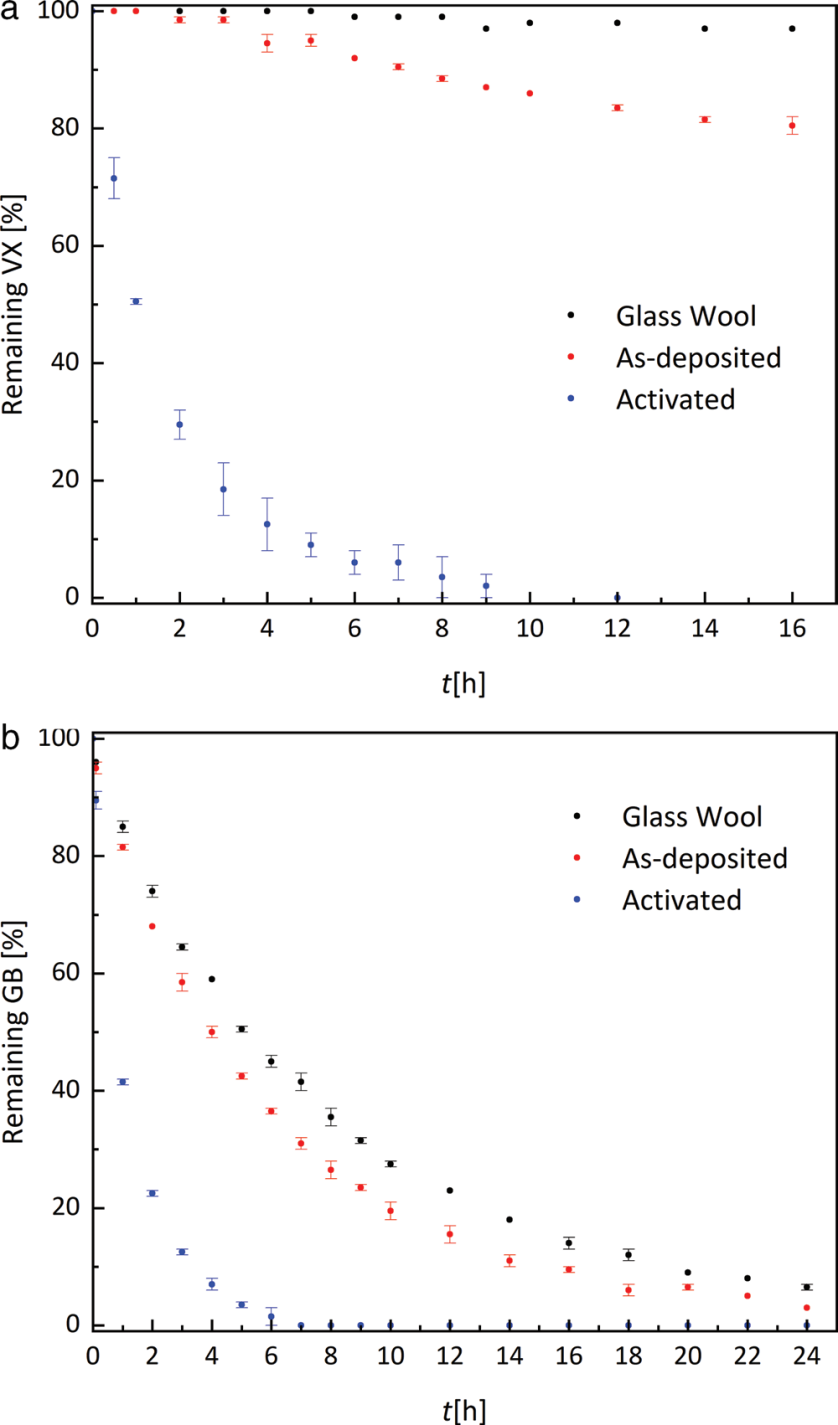
Degradation of a) VX and b) GB in MOPS buffer pH = 7.5 in the presence of glass wool, as‐deposited samples, or activated samples.

Finally, the catalytic properties of the MOF‐coated fibers were assessed by probing their activity towards degradation of the nerve agents VX and GB, which proceeds via hydrolysis.^[^ ^23^, ^32^ ^]^ Thus, a piece of the coated samples, as well as uncoated glass fibers were submerged in MOPS buffer pH = 7.5 containing the nerve agent, while the progress of agent hydrolysis was monitored by NMR spectroscopy. A neutral buffer was chosen instead of the more commonly employed *N*‐ethylmorpholine (NEM) buffer at pH = 10, as a first step approximation towards a neutral, solvent‐free environment. **Figure** **5**a shows the degradation curves of VX (1 mM) for the as‐deposited and scCO_2_ activated sample, as well as the uncoated glass wool as a reference. As expected, the as‐deposited sample (*t*_1/2_ ≈ 46 h) and the glass wool sample were virtually inactive. However, an approximately 75‐fold increase in hydrolytic activity was observed with the scCO_2_ activated MOF‐composite (*t*_1/2_ = 0.6 h) sample that completely degraded VX within 9 h. In case of GB, a 4‐fold rate increase was achieved, with GB being completely hydrolyzed with a half‐life of approximately 1.1 h (Figure 5b). The degradation observed with the as‐deposited sample (t_1/2_ = 4.3 h) can be mainly ascribed to spontaneous hydrolysis of the more labile sarin (*t*_1/2_ = 5.5 h). The agent loss of by adsorption and subsequent false indication of degradation was previously ruled out by previously conducted ^31^P NMR experiments with an external standard (a glass insert containing trimethylphosphate),^[^ ^23^ ^]^ where the concentration of agent and its degradation products in the solution were calculated based on the known concentration of the standard. No significant adsorption of agent (GD nor VX) to UiO‐66‐NH_2_ was detected.

**Figure 6:**
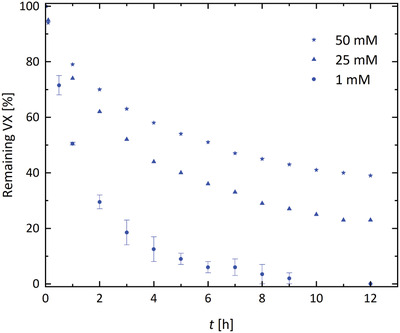
Degradation of VX in solutions of varying concentrations by the crystallized sample in MOPS buffer pH = 7.5.

In order to further challenge the performance of the fibers, the amount of VX in the solution was increased from 1 mM to 25 and 50 mM, respectively (**Figure** **6**). These experiments also led to degradation of the majority of VX within 12 h, albeit incomplete, which was presumably caused by a catalyst poisoning over time by the VX degradation products. Nonetheless, 20–30 µmol of VX was hydrolyzed by the estimated amount of 0.9 µmol of MOF, clearly demonstrating the catalytic nature of the reaction. These results also indicate that even a relatively low MOF loading is sufficient to catalyze the reaction thanks to the extended surface area.

## 3 Conclusion

We have successfully applied an all‐gas phase MLD process to deposit amorphous Zr‐MOF films on glass fiber substrates. These films were subsequently crystallized by a mild treatment in acetic acid to form the UiO‐66‐NH_2_ structure. It was found that the coated fibers were capable of both toxic gas retention and catalytic degradation of CWA, even though the MOF loading is as low as 11.5 wt%.

The crystallized samples showed much better performance than the as‐deposited ones in both adsorption and degradation experiments. The breakthrough experiments revealed that the NH_3_ adsorption capacity of the MOF‐covered glass fibers was lower than the previously reported values for bulk UiO‐66‐NH_2_. This may be ascribed to an incomplete conversion of the as‐deposited film to crystalline MOF material. Another important factor is the overestimation of the MOF loading caused by loss of MOF film during handling, as seen in SEM images.

In case of nerve agent degradation, the crystallization of the Zr‐MOF films resulted in a massive increase in their catalytic performance in the hydrolysis of VX and GB. It is worth mentioning that the degradation experiments were performed at neutral pH conditions to more accurately simulate a future buffer‐free, neutral environment. Thus, even higher catalytic activity may be expected under basic conditions. However, the aforementioned overestimation of the MOF loading and the ratio of crystalline to amorphous material prevents a direct comparison between our materials and bulk MOF samples. In this respect, the rate of the VX degradation was rather outstanding. We believe that the nanoscopic dimensions of the crystallites in the MOF film improve accessibility of the pores compared to that of bulk MOF materials, making the MOF thin films even more desirable for use as barrier materials in IPE.

The post‐synthetic treatment substantially improves the desired properties of the MOF films, however, it adds an extra step to the synthesis. Therefore, the future work should include targeting of higher quality crystal structures via modified MLD process, thus eliminating the need for post‐synthetic treatment. Although further optimizations are needed, the results demonstrate that MOF‐functionalization of fibrous materials for filter technology and protective clothing by MLD may be a promising route to develop IPE capable of both capturing and degradation of CWAs and TICs.

## 4 Experimental Section

### MOF Thin Films Deposition and Preparation

The thin film coatings were deposited on glass wool fibers (as‐received from Merck (Sigma Aldrich), Supelco, CAS number 65997‐17‐3, melting point 730 °C ) using the MLD method previously reported for flat Si substrates.^[^ ^41^ ^]^ During the deposition process the films thickness was monitored in situ by quartz crystal microbalance (QCM).^[^ ^41^ ^]^ Additionally, two Si substrates were placed in the MLD reactor together with the glass wool piece (one upstream from the glass wool and one downstream) for thickness measurement using the spectroscopic ellipsometry (SE). The deposition was performed by exposing the pieces of glass wool (≈ 8 × 5 × 2 cm^3^) to vapors of the MOF precursors 2‐amino‐1,4‐benzene dicarboxylic acid (H_2_‐2‐amino‐1,4‐BDC, Sigma‐Aldrich 99%) and zirconium (IV) tetrachloride (ZrCl_4_, MERCK Schuchardt OHG >98%) in an F‐120 Sat‐type ALD reactor (ASM Microchemistry Ltd). The following pulse and purge sequence was used: 10s ZrCl_4_ pulse, 5s purge, 8s H_2_‐2‐amino‐1,4‐BDC pulse, 6s purge. The samples were made with 500 cycles of this sequence with a reaction temperature of 265 °C. N_2_ (AGA 99.999%) was used for purging and as a carrier gas at the total flow of *≈*500 sccm (standard cubic centimeters per min) throughout the experiments, leading to a background pressure of *≈*5 mbar. The ZrCl_4_ and H_2_‐2‐amino‐1,4‐BDC precursors were heated to 165 and 225 °C, respectively, to achieve sufficient vaporization. The as‐deposited samples are referred to as “as‐deposited” throughout this paper.

In the previous work, the as‐deposited Zr‐MOF films synthesized by MLD were found to be amorphous, and their structural properties greatly improved upon treatment with acetic acid vapor,^[^ ^40^ ^]^ which led to conversion of the amorphous coating to crystalline MOF. Since the crystallinity of MOF materials influences their gas adsorption capacity and catalytic properties, it was decided to further examine the effects of this post‐synthetic treatment on the application‐related properties of the films (TIC adsorption and CWA degradation). A part of the as‐deposited sample was exposed to the previously described treatment, that is, they were heated to 160 °C for 24 h in a sealed autoclave (35 cm^3^) with *≈*0.1 cm^3^ acetic acid (MERCK KGaA 100%). These samples are referred to as “crystallized samples”.

A supercritical CO_2_ (scCO_2_) activation step, similar to the method previously described in detail by the Farha group,^[^ ^50^ ^]^ was performed on some of the crystallized MOF films. Activation is an important step for removing adsorbed species from the MOF pores, giving rise to a significant improvement to the surface area. This, in turn, enhances the adsorption capacity and catalytic activity of the MOF.^[^ ^50^, ^51^ ^]^ A Tousimis critical point dryer (SAMDRI‐PVT‐3D) was used. The sample was submerged in ethanol and placed in the chamber, where liquid CO_2_ was used to purge the ethanol. After a complete replacement of the ethanol, the sample was heated until the CO_2_ reached its super critical state. The CO_2_ was then slowly released from the chamber (≈5 cm^3^ min^−1^) overnight. The crystallized samples that were activated in scCO_2_ are referred to as “activated samples”.

### MOF Thin Films Structural and Chemical Characterization

Spectroscopic ellipsometry (SE) was performed with a J.A. Woollam alpha‐SE spectroscopic ellipsometer over a wavelength range of 390–900 nm. The data were collected for the films on Si substrates and modeled in the CompleteEASE software package using a Cauchy‐function, in order to determine the thickness of these films. The average value of the measured thicknesses on the Si substrates from the front and the back of the reaction chamber was used as an estimate of the film thickness on the glass wool fibers.

X‐ray diffraction (XRD) was performed with a Bruker AXS D8 Discover diffractometer equipped with a LynxEye strip detector and a Ge (111) focusing monochromator, providing Cu *K_α_*_1_ radiation.

Scanning electron microscopy (SEM) images were obtained using a HITACHI SU6600 scanning electron microscope equipped with a Schottky FE electron gun. The acceleration voltage was typically 1 kV with a beam current of 10‐20 µA, and the working distance was approximately 30–35 mm.

A Bruker Vertex 70v with DTGS detector, equipped with a Bruker Platinum ATR cell was used to collect the infrared spectra. Measurements were made with a resolution of 16 cm^−1^ in the range of 4000–500 cm^−1^.

N_2_ adsorption isotherms were measured on a Micromeritics ASAP2020+ instrument. The samples obtained after scCO_2_ activation were used for these measurements after heating (50 ^o^C) in dynamic vacuum at 10 mm Hg for 16 h.

### Ammonia Capturing

**Figure 7:**
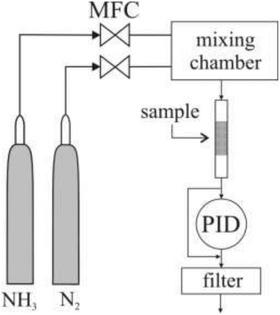
Schematic representation of the test rig used for measuring the breakthrough times of the MOF‐coated glass fibers.

Breakthrough time measurements under dynamic conditions were performed using glass sorption tubes (inner diameter = 4 mm, Supelco–), and a micro breakthrough test rig shown schematically in **Figure** **7**. The tubes were filled with approximately 15–20 mg of the MOF‐coated glass wool. The gas flow was controlled by a set of mass flow controllers (MFC, Bronkhorst). Prior to the measurement, the filled tubes were flushed with N_2_ (Linde HiQ, 6.0) for about 5 min before the challenge mixture (mixing chamber from Bronkhorst) of 200 ppm NH_3_ (Linde HiQ, 5.0) in N_2_ was applied with a steady flow of 20 cm^3^ min^−1^. The experiment was run under dry conditions (relative humidity < 2%), and at ambient temperature (*≈*22 °C). The NH_3_ concentration in the effluent gas was monitored using a photoionization detector (PID, PhoCheck Tiger, Ion Science) with a 10.6 eV lamp. Ideally, the breakthrough should be monitored until the effluent concentration reaches maximum value, and the adsorption capacity then calculated by integrating the breakthrough curve. However, it is found that at higher NH_3_ concentrations the PID response showed some sensitivity loss due to prolonged exposure to concentrated NH_3_. Since this could affect the results recorded in this concentration range, it was decided to calculate the adsorption capacity at the breakthrough time (*A*_bt_). The breakthrough time was achieved when the NH_3_ concentration in the effluent reached 10% of the influent level (i.e., 20 ppm), that is above the mean odor detector threshold (ODT,^[^ ^52^ ^]^), yet below the permissible exposure limit (PEL, e.g. in USA NH_3_ PEL is 25 ppm averaged over 8 h period^[^ ^53^ ^]^).

### Degradation of VX and GB

CAUTION: VX and sarin (GB) are extremely toxic agents and should only be used by trained personnel in a facility that has the permission to work with these agents.

Changes in agent concentrations were measured using Nuclear Magnetic Resonance (NMR) spectroscopy on a Bruker Avance III NMR Spectrometer, with a broadband probe, operating at 400 MHz (^1^H). Solutions of either VX or GB (1, 25 or 50 mM) were prepared in 3‐morpholinopropane‐1‐sulfonic acid (MOPS) buffer (0.1 M pH = 7.5, in 10% D_2_O/H_2_O). The 25 and 50 mM solutions were prepared b**y** weighing the appropriate amount of agent in an NMR tube and subsequently adding 1 cm^3^ of MOPS buffer (0.1 M pH 7.5, in 10% D_2_O/H_2_O). The 1 mM solution was prepared by dilution of 100 µL of a 10 mM stock solution of VX or GB in acetonitrile with 900 µL of MOPS buffer in an NMR tube (5 mm). The (functionalized) glass wool (14 mg) was cut and pushed through the solution to the bottom of the NMR tube with a long glass pipette, making sure that any air trapped between the fibers was squeezed out of the wool plug using the pipette tip. The 25 and 50 mM experiments were analyzed by running a series of standard ^31^P NMR measurements and integrating the VX (61 ppm) and ethyl methylphosphonic acid (EMPA, 27 ppm) signals (the sum of which was denoted as 100%).

The 1 mM experiments were analyzed using a series of 1D ^31^P‐^1^H heteronuclear single quantum coherence (HSQC) measurements, using d1 = 1s, ns = 32‐64, and X = 16 Hz.^[^ ^54^, ^55^ ^]^ The doublet peaks at 1.85–1.95 ppm (VX), 1.65–1.8 ppm (GB), 1.1–1.3 ppm (isopropyl methylphosphonic acid (IMPA)), and 1.15–1.30 ppm (EMPA) were integrated. The sum of the integrals was denoted as 100%.

## Conflict of Interest

The authors declare no conflict of interest.

## Supporting information

Supporting Information

## Data Availability

The data that support the findings of this study are available from the corresponding author upon reasonable request.
