# Supplementary material for: Utilizing Zirconium MOF‐functionalized Fiber Substrates Prepared by Molecular Layer Deposition for Toxic Gas Capture and Chemical Warfare Agent Degradation
Source: Glob Chall. 2021 Sep 12;5(12):2100001. doi: 10.1002/gch2.202100001 (PMC8671619; doi:10.1002/gch2.202100001)
Supplement: Supplementary file 1 — Supporting Information [file GCH2-5-2100001-s001.pdf]

© 2021 Wiley-VCH GmbH

Global Challenges Open Access

Supporting Information

for *Global Challenges,* DOI: 10.1002/gch2.202100001

Utilizing Zirconium MOF-functionalized Fiber Substrates Prepared by Molecular Layer Deposition for Toxic Gas Capture and Chemical Warfare Agent Degradation

Agnieszka Gorzkowska-Sobas, Kristian Blindheim Lausund, Martijn C.de Koning, Veljko Petrovic, Sachin M. Chavan, Martin W. Smith, and Ola Nilsen*

Supporting Information

Utilizing Zirconium MOF-functionalized fiber substrates prepared by molecular layer deposition (MLD) for toxic gas capture and chemical warfare agent degradation

Agnieszka Gorzkowska-Sobas, Kristian Blindheim Lausund, Martijn C. de Koning, Veljko Petrovic, Sachin M. Chavan, Martin W. Smith, Ola Nilsen*

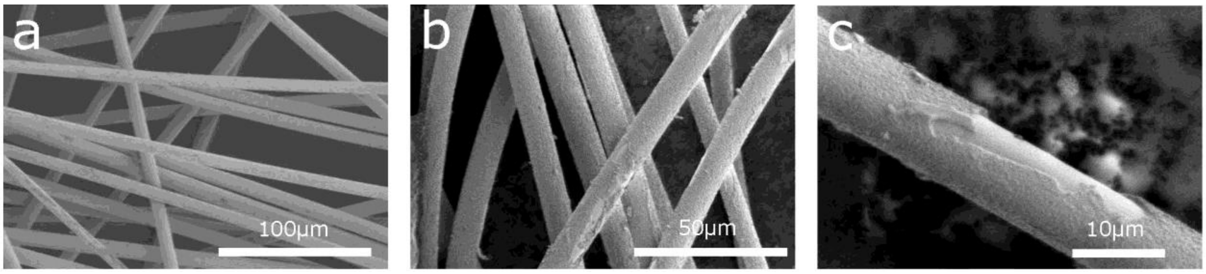

Figure S1. SEM images of the glass fibers surface after MOF deposition a) as-deposited, b) crystallized. Some lose flakes of the material that has partially delaminated from the fibers can be seen, c) crystallized film showing partially exposed glass fiber.

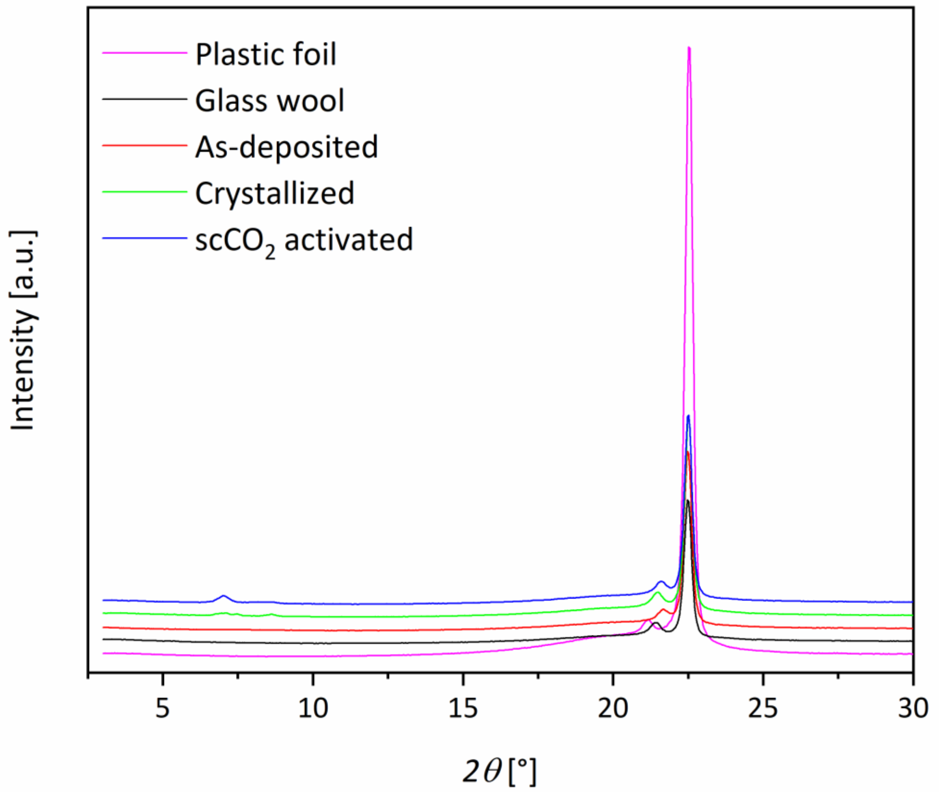

Figure S2. XRD diffraction pattern for the samples and the uncoated glass wool. The patterns reveal weak intensity reflections for MOF within the 5-10°, and strong reflections within 20-25° from the plastic foil used for confining the samples to the holder. An empty holder with plastic foil and an uncoated glass wool sample were measured for reference, and show no reflections at low angles that would interfere with the expected reflections from UiO-66-NH_2.

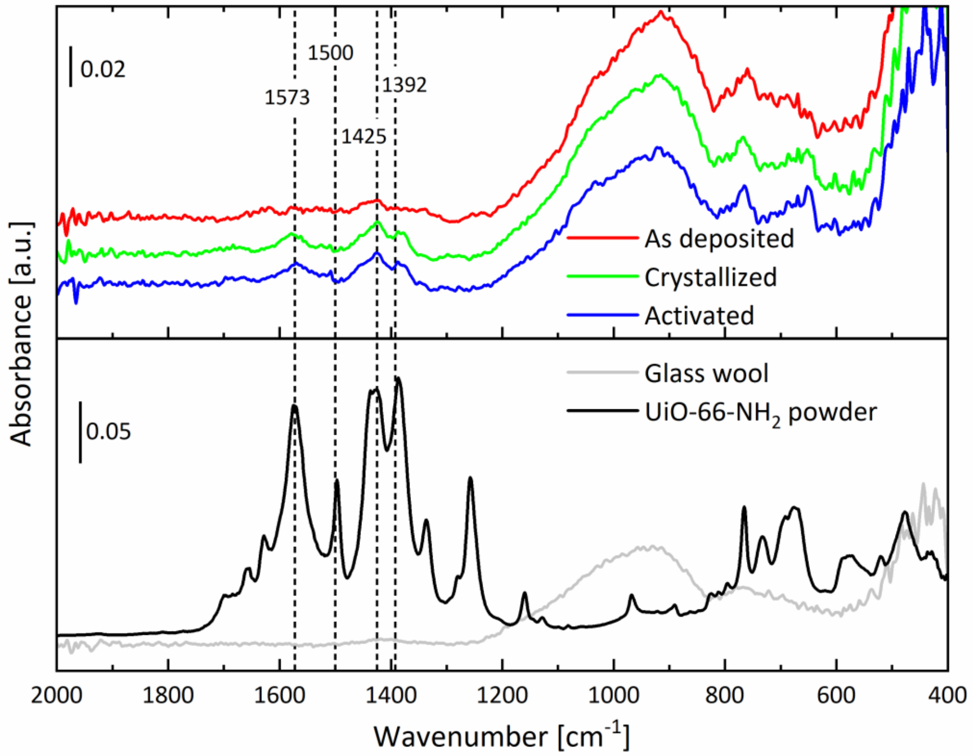

Figure S3. FTIR spectra for as-deposited, crystallized and activated samples (top) compared to uncoated glass wool and bulk UiO-66-NH_2 powder for reference (bottom). The spectra show vibrational peaks associated with a bidentate linker coordination with the wavenumber values for carboxylate asymmetric stretch (1573 cm^{-1}) and symmetric stretch (1425 cm^{-1}) (i.e. the splitting between the symmetric and asymmetric stretches of the carboxylate group is in the expected range), as well as the features seen in the carboxylate region (1350-1650 cm^{-1}) correspond well with those observed for bulk UiO-66-NH_2 [1]. The spectrum for the glass fibers coated with as-deposited films showed none of the sharp peaks expected for crystalline UiO-66-NH_2, revealing that the sample was amorphous. However, weak absorption features in the 650-800 cm^{-1} region and at 1255 cm^{-1} associated with UiO-66-NH_2 are present for all the coated fibers, and absent in the uncoated glass wool.

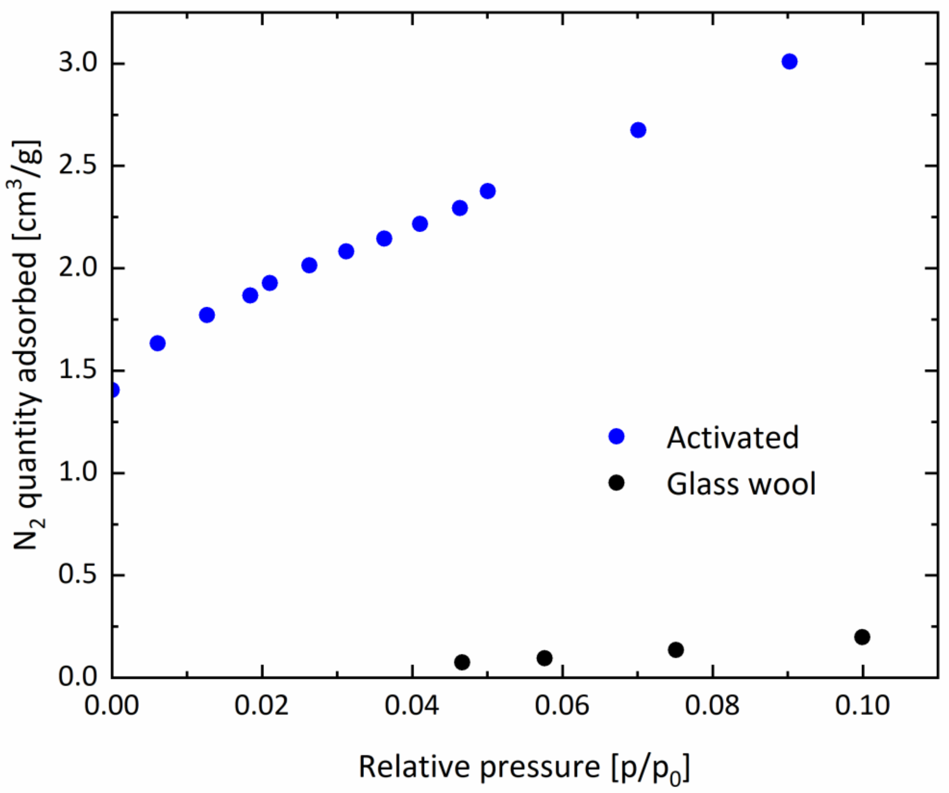

Figure S4. Nitrogen adsorption isotherm for the MOF/glass fiber composite and an uncoated glass wool reference.

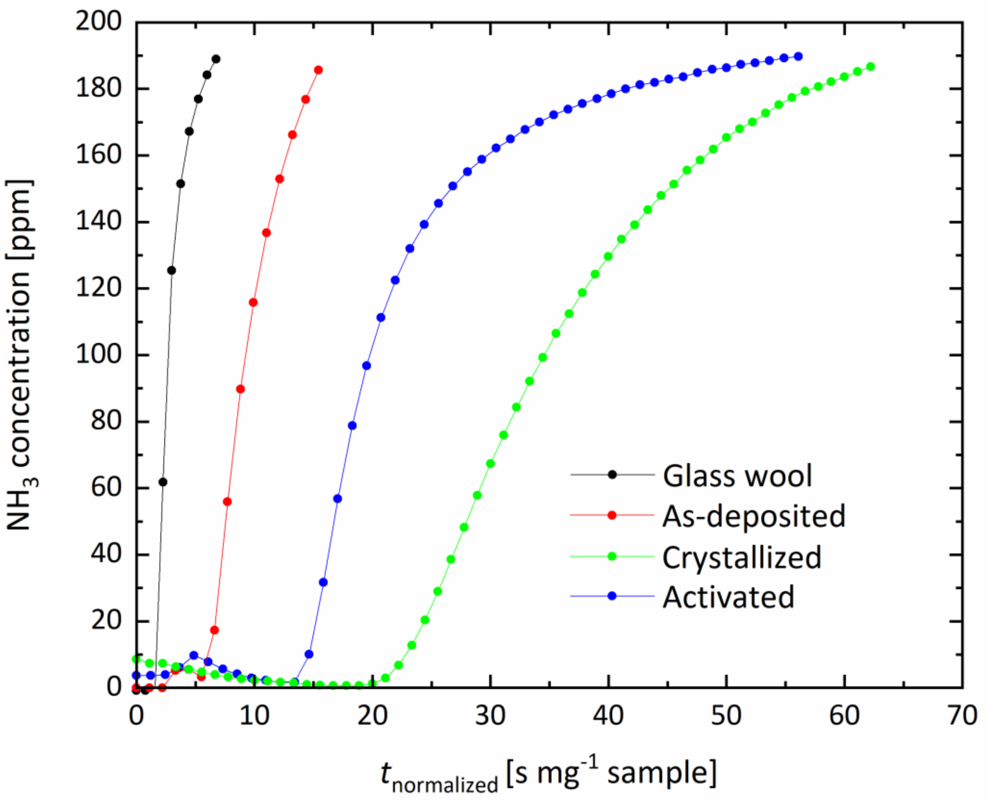

Figure S5. NH_3 breakthrough curves for uncoated and MOF coated glass wool samples. Uncoated wool shows a retention time of approx. 3 seconds, caused mainly by a dead volume of the experimental setup (this delay time was subtracted from the A_{bt} data obtained for the coated samples in Table 1).

References

[1] S. M. Chavan, G. C. Shearer, S. Svelle, U. Olsbye, F. Bonino, J. Ethiraj, K. P. Lillerud, S. Bordiga, *Inorganic Chemistry* **2014,** 18, 9509
